# A Novel Low-Power-Consumption All-Fiber-Optic Anemometer with Simple System Design

**DOI:** 10.3390/s17092107

**Published:** 2017-09-14

**Authors:** Yang Zhang, Fang Wang, Zhihui Duan, Zexu Liu, Zigeng Liu, Zhenlin Wu, Yiying Gu, Changsen Sun, Wei Peng

**Affiliations:** School of Physics and Optoelectronic Technology, Dalian University of Technology, 2 Linggong Road, Ganjingzi District, Dalian 116024, China; yangzhang@dlut.edu.cn (Y.Z.); w15122514950@163.com (F.W.); 18844138725@163.com (Z.D.); liuzexu@dlut.edu.cn (Z.L.); ily.lzg@foxmail.com (Z.L.); zhenlinwu@dlut.edu.cn (Z.W.); yiyinggu@dlut.edu.cn (Y.G.); suncs@dlut.edu.cn (C.S.)

**Keywords:** fiber-optic sensors, tilted fiber Bragg grating, carbon nanotube, flow diagnostics

## Abstract

A compact and low-power consuming fiber-optic anemometer based on single-walled carbon nanotubes (SWCNTs) coated tilted fiber Bragg grating (TFBG) is presented. TFBG as a near infrared in-fiber sensing element is able to excite a number of cladding modes and radiation modes in the fiber and effectively couple light in the core to interact with the fiber surrounding mediums. It is an ideal in-fiber device used in a fiber hot-wire anemometer (HWA) as both coupling and sensing elements to simplify the sensing head structure. The fabricated TFBG was immobilized with an SWCNT film on the fiber surface. SWCNTs, a kind of innovative nanomaterial, were utilized as light-heat conversion medium instead of traditional metallic materials, due to its excellent infrared light absorption ability and competitive thermal conductivity. When the SWCNT film strongly absorbs the light in the fiber, the sensor head can be heated and form a “hot wire”. As the sensor is put into wind field, the wind will take away the heat on the sensor resulting in a temperature variation that is then accurately measured by the TFBG. Benefited from the high coupling and absorption efficiency, the heating and sensing light source was shared with only one broadband light source (BBS) without any extra pumping laser complicating the system. This not only significantly reduces power consumption, but also simplifies the whole sensing system with lower cost. In experiments, the key parameters of the sensor, such as the film thickness and the inherent angle of the TFBG, were fully investigated. It was demonstrated that, under a very low BBS input power of 9.87 mW, a 0.100 nm wavelength response can still be detected as the wind speed changed from 0 to 2 m/s. In addition, the sensitivity was found to be −0.0346 nm/(m/s) under the wind speed of 1 m/s. The proposed simple and low-power-consumption wind speed sensing system exhibits promising potential for future long-term remote monitoring and on-chip sensing in practical applications.

## 1. Introduction

Fiber-optic sensors have become attractive candidates for various kinds of measurements, such as temperature, axial strain, and acoustic emission, due to their distinguished advantages of small size, high accuracy, anti-electromagnetic interference, long-term stability, and multiplexing capability [1,2,3,4,5]. As the demand for compact and long-term remote wind sensing systems has rapidly increased, more and more anemometers have been investigated in recent years. Most of them were traditional electronic sensors, including volumetric, turbine, differential pressure, ultrasonic, and electromagnetic flowmeter [6,7,8,9,10]. Recently, with the development of optical fiber sensors, many fiber-optic anemometers have been proposed, and great efforts have been carried out to achieve simple and compact sensing systems with low-cost and enhanced efficiency, especially for long-term remote sensing. 

Among them, hot-wire fiber anemometer (HWA) is an important type that has been widely investigated [11,12]. The basic principle of the well-known HWA is that, in general, as the sensor is heated up, the speed of wind flow around the HWA can be calculated by measuring the cooling rate or temperature variation of the sensor head [13]. The same ideas have been applied to the fiber-optical wind speed sensing. The initial reported heating methods through fibers were to guide an input pump laser to interact with metallic film on the fiber surface. Geometry-modified fiber structures were proposed, including bending, tapering, making bubbles in the fiber, and mismatch fusing of multimode fiber with single mode fiber [14,15,16,17]. Usually, a fiber Bragg grating (FBG) was applied to measure the temperature near the heating region. However, these methods will significantly weaken the strength of the sensor structure. Facing this problem, Caldas et al. proposed a fiber-optic hot-wire flowmeter based on a metallic coated hybrid long period fiber Bragg grating (LPG) structure [18]. They used an LPG to couple laser light in the core to interact with the Ag film coated near the LPG region, and another FBG was inscribed under the coating to measure the temperature variation. Although this design avoided the geometry modification of the fiber, the sensing structure was still complicated and showed low light-heat conversion efficiency. Gao et al. reported a fiber-optic thermal anemometer via cobalt-doped fiber with FBG [19]. The anemometer utilized the cobalt-doped fiber in the sensor to absorb light efficiently and heat an FBG to be a “hot-wire” instead of metal coating. The structure of this sensor was further simplified, but it still needed a special high-power laser with a certain wavelength of 1450 nm to heat the FBG. The inscription of FBG in special Co^2+^-doped fiber is also hard and expensive, which will raise the cost. Besides, for long-term sensing in practice, power consumption is a key and inevitable issue that needs to be taken into consideration, and so far, few efforts have been devoted to this issue. Most recently, Wang et al. proposed an interesting low-power-consumption fiber-optic anemometer based on a metal-filled microstructured optical fiber (MOF) and FBG [20]. A special six-hole MOF was designed and fabricated for FBG inscription and metal infiltration. The developed metal-filled fiber anemometer exhibits great light–heat conversion efficiency with a significantly low pumping power of less than 10 mW. This work dealt well with the power consumption problem. However, in order to lower the power consumption, the simplicity and strength of the sensor structure were sacrificed. The fabrication complexity of the sensor reduces the practicability and raises the cost. Furthermore, it still needs one laser to heat the FBG and another broadband light source (BBS) as the sensing light source. Therefore, it is a fundamental challenge to design a simple low-cost wind speed sensing system with a compact structure and lower power-consuming properties.

In this paper, a novel fiber wind speed sensor with a compact size and low power consumption is proposed, which is based on single-walled carbon nanotubes (SWCNTs) coated titled fiber Bragg grating (TFBG). TFBG is a kind of fiber Bragg grating (FBG) with the gratings tilted with respect to the fiber axis. As a novel near-infrared wavelength sensing element, it possesses unique capability of easily coupling the broadband lights propagating in the core to the fiber surface without any geometry change of the fiber [21,22,23,24]. Using a single TFBG, the coupling and sensing functions of the proposed anemometer can be achieved simultaneously. To further enhance the absorption efficiency of the sensor, single-walled carbon nanotubes (SWCNTs) are used as the key thermal conversion medium immobilized on the fiber surface. SWCNTs are innovative nanomaterials with excellent infrared light absorption capability and much higher thermal conductivity compared with traditional metallic materials such as silver. A compact sensor head is easily fabricated after the SWCNT film is coated on the TFBG. The combination of the SWCNTs and TFBG with great coupling and absorbing capability can significantly reduce the required pumping power. Thus, to lower the power consumption and the cost of the whole sensing system, no extra laser-pumping source is needed and only one BBS is applied as both the heating and sensing source. In the experiments, the key parameters of the sensor, including the film thickness and the inherent angle of the TFBG were fully investigated. The proposed simple and low-power-consumption anemometer system exhibits promising potentials for future long-term remote sensing in practical applications. 

## 2. Principle and Experimental System

**Figure 1:**
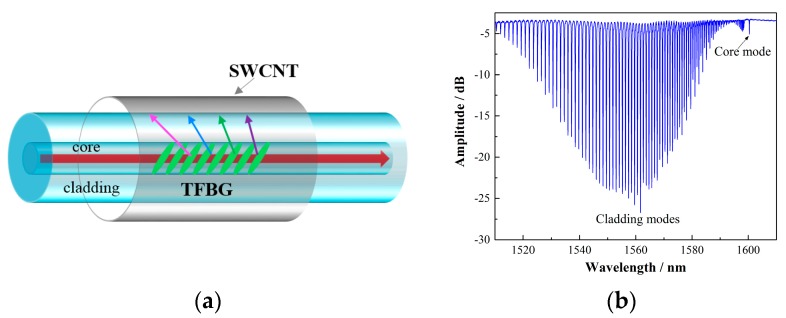
(**a**) Schematic diagram of the wind speed sensor; (**b**) spectrum of a common TFBG. TFBGs are a special kind of fiber Bragg grating, where the gratings are tilted with respect to the fiber axis and can couple light from the core to the cladding.

The schematic configuration of the proposed sensor head is shown in Figure 1a. The TFBG was written in hydrogen-loaded common Corning SMF-28 fiber via a 248 nm UV excimer laser and phase-mask method. During the fabrication, the laser pulse was set to be 6 mJ/150 Hz, and the TFBG was made by a scanning technique. Figure 1b presents a typical transmission spectrum of the TFBG. It clearly shows that a number of cladding mode resonances are excited by the TFBG. To deposit SWCNTs on the fiber surface with controllable process and low cost, a dip-coating method was used. Ten milligrams of carboxylic SWCNTs were dispersed in a DMF (*N*,*N*-dimethylformamide) solution using sonication. Then, the TFBG was dipped into a 0.1 wt % aqueous APTES (3-aminopropyltriethoxysilane) solution for 1 min to produce amino-terminated (silanized positively charged surface). After that, the functionalized TFBG was submerged in the DMF-CNT suspension for 30 s. The positively charged fiber surface will readily immobilize the negatively charged and functionalized SWCNTs. The dipping cycle was repeated to increase the thickness of the nanotube layer gradually. 

For a TFBG, its Bragg wavelength $\lambda_{Bragg}$ (nm) and ith cladding mode resonance wavelength $\lambda_{cladding}^{i}$ (nm) are determined by the following phase matching conditions:

(1) $$\lambda_{Bragg}=2n_{effcore}\Lambda /\cos(\theta )$$

(2) $$\lambda_{cladding}^{i}=(n_{effcore}+n_{cladding}^{i})\Lambda /\cos(\theta )$$

where Λ (nm) is the period of the TFBG, θ (degree) is the tilt angle of the grating planes relative to the plane of the fiber cross section, and $n_{effcore}$ (dimensionless) and $n_{cladding}^{i}$ (dimensionless) are the effective refractive index of the fiber core and cladding. 

The proposed sensor is based on the principle of hot-wire anemometry. HWA is a well-known technique for wind speed measurement, which is based on the heat transfer from sensors to the surrounding environment. In general, when a dielectric film is heated up, the speed of wind flow around the HWA determines the cooling rate of the film. Based on the theory of hot-wire anemometry [13], the relationship between the heat loss $H_{\mathrm{loss}}$ (J) and the wind speed ν (m/s) can be expressed as follows:

(3) $$H_{\mathrm{loss}}=\Delta T\cdot (A+B\sqrt{\nu })$$

where ΔT (degree Centigrade) is the temperature change of the sensor, and A and *B* are empirical calibration constants. When we use the TFBG to measure the temperature, the core and *i*th cladding mode wavelength shifts ($\lambda_{Bragg}$, $\lambda_{cladding}^{i}$) caused by temperature change (ΔT) can be expressed as follows:

(4) $$\Delta \lambda_{Bragg}=(2\frac{n_{effcore}}{\cos(\theta )}\frac{d\Lambda }{dT}+2\frac{\Lambda }{\cos(\theta )}\frac{dn_{effcore}}{dT})\Delta T$$

(5) $$\Delta \lambda_{cladding}^{i}=(\frac{\left(n_{effcore}+n_{cladding}^{i}\right)}{\cos(\theta )}\frac{d\Lambda }{dT}+\frac{\Lambda }{\cos(\theta )}\frac{d(n_{effcore}+n_{cladding}^{i})}{dT})\Delta T.$$

By substituting Equation (3) in Equations (4) and (5), the wavelength of the core mode and cladding modes can be expressed as a function of wind speed:

(6) $$\lambda_{Bragg}=\lambda_{Bragg0}+(2\frac{n_{effcore}}{\cos(\theta )}\frac{d\Lambda }{dT}+2\frac{\Lambda }{\cos(\theta )}\frac{dn_{effcore}}{dT})\frac{H_{loss}}{\left(A+B\sqrt{\nu }\right)}$$

(7) $$\lambda_{cladding}^{i}=\lambda_{cladding0}^{i}+(\frac{\left(n_{effcore}+n_{cladding}^{i}\right)}{\cos(\theta )}\frac{d\Lambda }{dT}+\frac{\Lambda }{\cos(\theta )}\frac{d(n_{effcore}+n_{cladding}^{i})}{dT})\frac{H_{\mathrm{loss}}}{\left(A+B\sqrt{\nu }\right)}$$

where $\lambda_{Bragg}$ and $\lambda_{Bragg0}$ are the instant and initial Bragg wavelength, respectively. Additionally, $\lambda_{cladding}^{i}$ and $\lambda_{cladding\;0}^{i}$ are the instant and initial ith cladding mode wavelength, respectively. From Equations (6) and (7), similar wavelength responses of the core mode and the cladding modes with respect to the wind speed should be expected, since the $n_{effcore}$ and $n_{cladding}^{i}$ have a very small difference.

**Figure 2:**
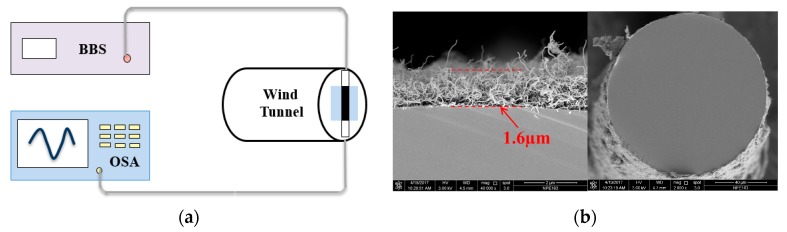
(**a**) Wind speed sensing system with only a BBS and a wavelength demodulation device. (**b**) SEM images of the SWCNT film on the fiber surface. The thickness of the film is 1.6 μm with 50 dipping cycles.

Figure 2a shows the experimental setup of the proposed wind speed sensing system. Only one C + L band (1528–1603 nm) BBS was used in the system to cover the TFBG spectrum and serve as both the sensing and heating source. TFBG has the special ability of coupling a broadband wavelength light (usually over 50~100 nm range depending on the tilted angle) to the cladding, which enhances the coupling efficiency compared with a single wavelength pumping laser light. As the light enters into the device, most of the light within the TFBG’s spectrum can be coupled into the higher order of cladding modes or the radiation modes and strongly absorbed by the SWCNTs deposited on the fiber surface. As can be seen from the SEM pictures in Figure 2b, here, SWCNTs are utilized as thermal conversion medium due to their competitive thermal characters. For the HWA design, the most interesting thing is that the SWCNT film is a strong infrared light absorber superior to the traditional metal materials. The thermal conductivity of SWCNTs is almost 10 times higher than that of silver. This absorption raises the local temperature of the fiber making the sensor heat a “hot wire.” When this “hot-wire” is put into the wind field, the heat will be taken away from the sensor head and the temperature variation can be accurately detected by the TFBG resonance peak shift. In the system, the wavelength response was monitored with an optical spectrum analyzer (OSA) with a minimum wavelength resolution of 0.02 nm. 

## 3. Results and Discussion

### 3.1. Temperature Responses

**Figure 3:**
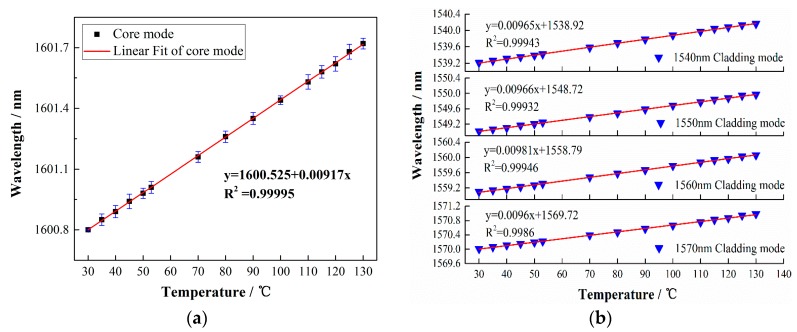
(**a**) The linear fit of wavelength response of the core mode under different temperatures. A 12° TFBG with a 1.6 µm coating was used. (**b**) The linear fit of cladding modes wavelength responses to temperature, including the cladding modes near 1540 nm, 1550 nm, 1560 nm, and 1570 nm.

**Figure 4:**
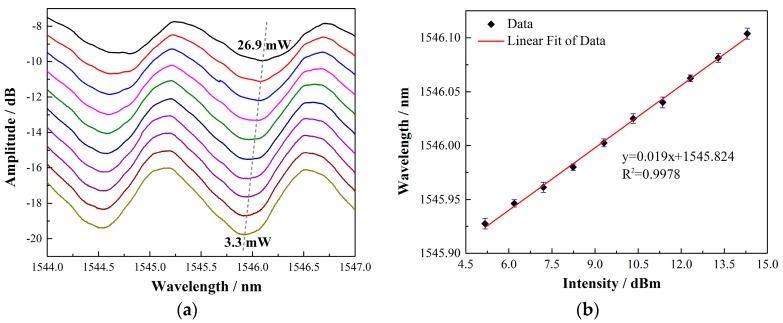
(**a**) Cladding modes evolution with different input powers. As the power increased, the wavelength showed a red shift. (**b**) The cladding mode wavelength shift with different input powers.

As the surrounding temperature changes, for a fiber Bragg grating, thermal expansion will cause the grating period change. In addition, the thermo-optic effect also has an impact on the refractive index of the fiber grating, as can be seen from Equations (6) and (7). The thermal response of the TFBG was measured by putting an SWCNT-coated TFBG in an electric furnace at a controllable temperature range between 30 and 140 °C. A 12° TFBG with a 1.6 μm SWCNT film was applied in the experiments. The temperature sensitivities of the core mode and cladding modes are shown in Figure 3. The slopes for the core mode and the cladding modes are 0.00965, 0.00966, 0.00981, and 0.0096, respectively. As expected, the temperature responses of the cladding modes and the core mode are almost the same. Furthermore, the maximum wavelength change under a certain BBS input power determines most of the sensor performances including the sensing range and even the sensitivity. To characterize the power conversion efficiency of the anemometer, the wavelength shifts with respect to different BBS input powers were detected, which is depicted in Figure 4a. The output power of broadband source increased gradually, from 3.3 to 26.9 mW (5.17–14.3 dBm), and the wavelength response showed a linear red shift with an efficiency of 0.019 nm/dBm, as can be seen from Figure 4b.

### 3.2. Measurement Results of the Anemometer

**Figure 5:**
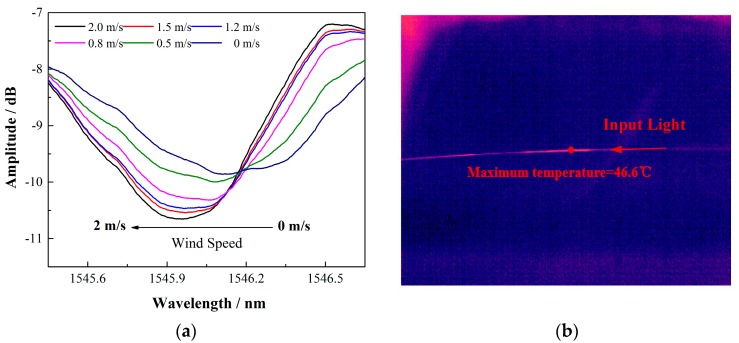
(**a**) The spectral response of one cladding mode under different wind speeds. Increasing the wind speed from 0 to 2 m/s results in a blue shift of the resonance from 1546.172 nm to 1545.952 nm. (**b**) The temperature distribution image detected by the MAG30 on-line thermal imager. The maximum temperature was measured up to be 46.6 °C when the input power was 22.97 mW.

With this highly simplified sensing system, the wind speed response using a 15 mm long 12° TFBG coated with a 1.6 μm thin SWCNT film was investigated first. The sensor was put into a small wind tunnel in which the wind speed could be tuned from 0 to 2.1 m/s (limited by our equipment) and recorded by an electrical anemometer (TESTO405V1). Figure 5 presents the spectral response under different wind speeds and temperature distribution image of the sensor via the MAG30 on-line thermal imager. In the experiments, the cladding mode around 1546 nm was selected to measure the temperature. As shown in Figure 5a, with a small input BBS power of 22.97 mW, the maximum wavelength shift is as much as 0.220 nm when the wind speed is increased from 0 to 2.0 m/s. Benefited from the significant coupling and absorbing efficiency of the proposed sensor, this response is comparable to that of the reported silver coating LPG/FBG anemometer and rare earth Co^2+^-doped fiber anemometer [18,19], while the input power of the proposed mechanism is almost one magnitude lower than that of the reported structures. It can also be seen in Figure 5b that the temperature of the local area on the TFBG was heated up to 46.6 °C under the input power of 22.97 mW. Thus, our proposed simple and low-cost sensing system proved promising. To further control and optimize the performances of the sensor, the key parameters, including the inherent angle of the TFBG, the coating thickness of the SWCNT film, and the input power of the BBS, were fully investigated. 

**Figure 6:**
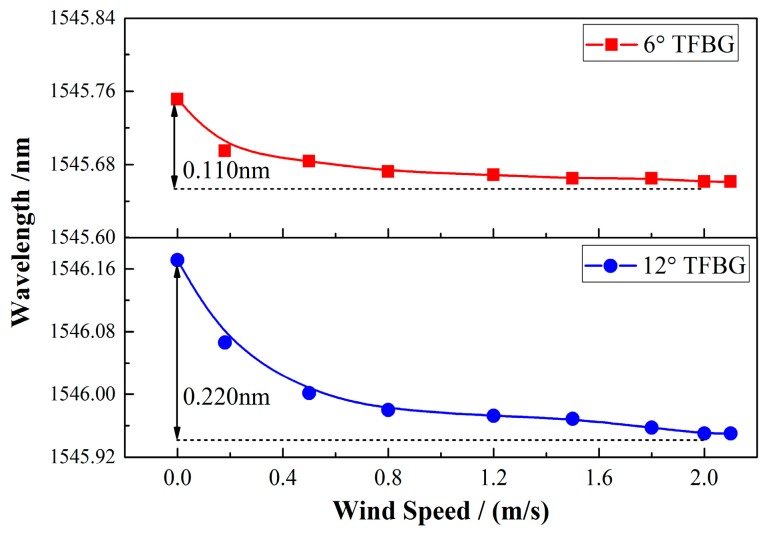
Comparison of the wind speed responses of the anemometer with different angles of TFBG under similar input powers and film thicknesses.

The main difference between a TFBG and a common FBG is the tilt angle between the grating plane and the fiber axis, which is the most important factor that distinguishes the spectral characteristics of a TFBG from an ordinary FBG. In principle, with a larger tilt angle, more lights propagating in the fiber core will be coupled to the higher-order cladding modes or converted to the radiation mode, which probably enhances the coupling efficiency. In the experiment, the wind speed responses of the anemometers fabricated by TFBGs with different angles were measured under the same input power (22.97 mW) and SWCNT film (1.6 μm). As shown in Figure 6, when the 6° TFBG was applied, the total wavelength change was measured to be 0.110 nm, which was only half of the wavelength change of 0.220 nm for the 12° TFBG. It is notable that both of the sensors have the same, nonlinear response as the wind speed, as expected from Equation (7). Hence, there is no doubt that the larger angle of the TFBG will offer an obvious boost to the sensitivity of the wind sensor.

**Figure 7:**
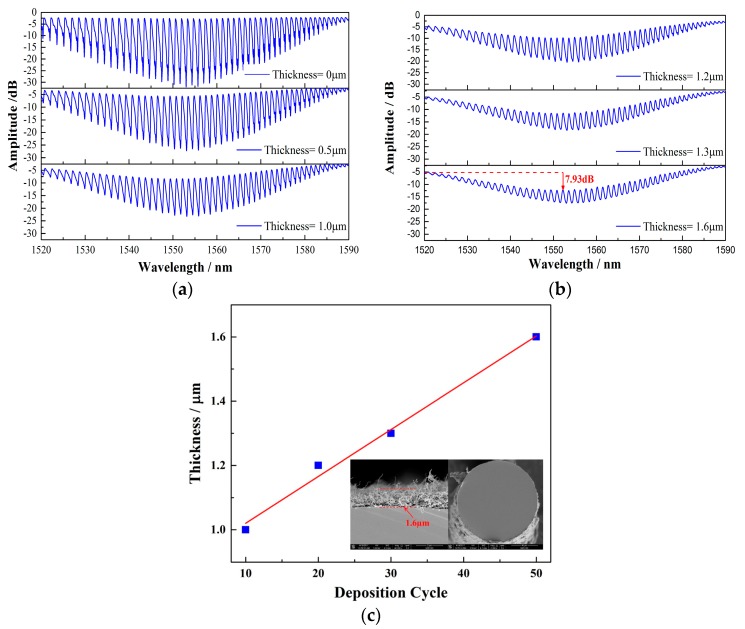
(**a**,**b**) The transmission spectrum of a 12° TFBG with different SWCNT film thicknesses. The depth of the upper envelope of the cladding modes are 0 dB, 1.001 dB, 5.312 dB, 6.758 dB, 7.372 dB, and 7.93 dB, respectively. (**c**) The SWCNT film thickness with respect to the deposition cycle.

SWCNTs are rolled up forms of graphene, a two-dimensional honey comb structure of carbon, and have exceptional electrical, optical, thermal, and mechanical properties [25,26,27]. It is the excellent thermal characteristics that make SWCNTs a good thermal conversion medium that is superior to traditional metal films such as silver. The fundamental interaction between the light coupled by the TFBG and the SWCNTs deposited on the fiber can be observed directly in the entire optical spectrum. The strong absorption will result in the spectrum compression of the cladding modes over a large wavelength range. In the experiment, the spectrums of the TFBG with different film thicknesses from 0 to 1.6 μm were investigated, as shown in Figure 7a,b. We can clearly see that, with the increase in the thickness, the transmission spectrum is compressed, indicating that more powers are coupled to the fiber surface and absorbed by the films. By monitoring the upper envelope of the cladding modes, the coupling or absorbing efficiency can be roughly estimated. As shown in Figure 7, the depth of the upper envelope of the cladding mode resonances are measured to be 0 dB, 1.001 dB, 5.312 dB, 6.758 dB, 7.372 dB, and 7.93 dB, respectively, which correspond to the film thicknesses of 0, 0.5 μm, 1.0 μm, 1.2 μm, 1.3 μm, and 1.6 μm. Therefore, with a thicker film, more lights in the fiber core can be absorbed by the immobilized SWCNT film on the fiber surface and converted to the heat raising the fiber temperature.

**Figure 8:**
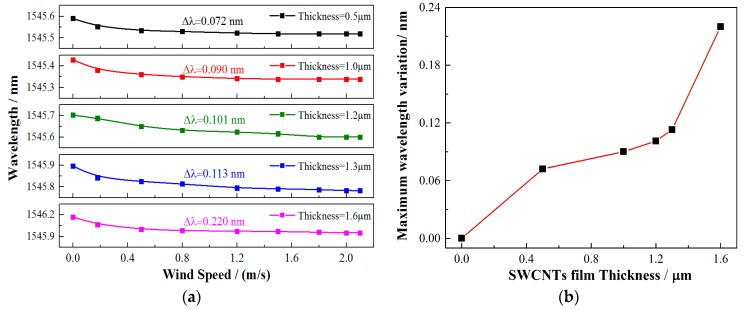
(**a**,**b**) Wavelengths responses of the 12° TFBGs with different film thicknesses under the same input power of 22.97 mW.

The wind speed response of the sensors with different coating thicknesses were then investigated. All the sensors were fabricated with the same inherent angle of 12° and measured under the same BBS input power of 22.97 mW. In Figure 8, the influences of coating thickness to the anemometer’s responses were depicted. For each sensor, the maximum wavelength shifts are 0.072 nm, 0.090 nm, 0.101 nm, 0.113 nm, and 0.220 nm, respectively, when the wind speed is tuned from 0 to 2 m/s. As a result, increasing the thickness of the SWCNT film is a simple and effective way to improve the sensitivity of the anemometer. However, considering the spectrum compression of the TFBG, which will affect the cladding modes’ visibility and sensing resolution, the film thickness should be optimized to a certain point. In our experiments, a 1.6 μm coating was preferred.

**Figure 9:**
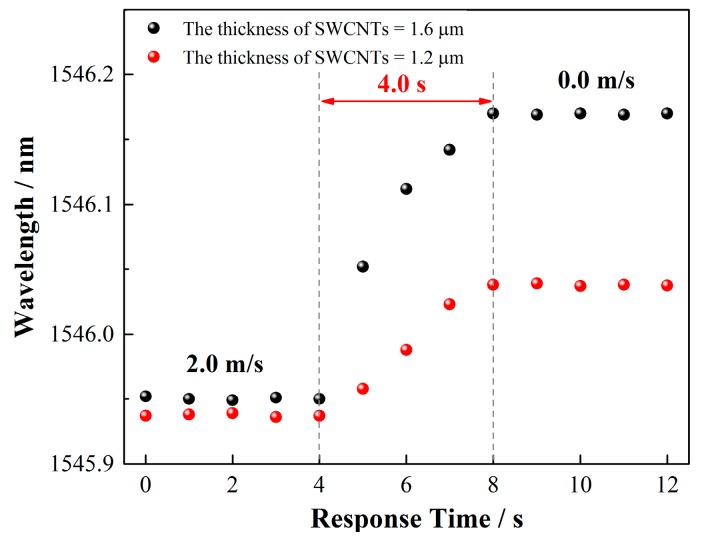
Sensitivity as a function of wind speed under different input powers.

The response time of the wind speed sensor is a key parameter and the temperature gradient in the SWCNT layer might affect the response time of the sensor to the wind. To evaluate this parameter and the influence, experiments were conducted to measure the response time of the sensor using a 1.6-μm-coated 12° TFBG. Figure 9 exhibited the results; a 4.0 s time response was obtained for the 1.6 μm coating as the wind speed varied from 2.0 to 0 m/s. Furthermore, the response time of the two sensors with different coating thicknesses of 1.6 μm and 1.2 μm was compared, and no obvious difference was observed for the response time. The thickness has little influence on the response time of the sensor, which might be a result of the larger thermal conductivity of the SWCNTs, which is 6600 W/(m·K) (almost 10 times that of the silver film). This result is important for coating thickness optimization of the sensor because the response time is not a concern when a thicker layer (within our interested thickness range) is applied.

**Figure 10:**
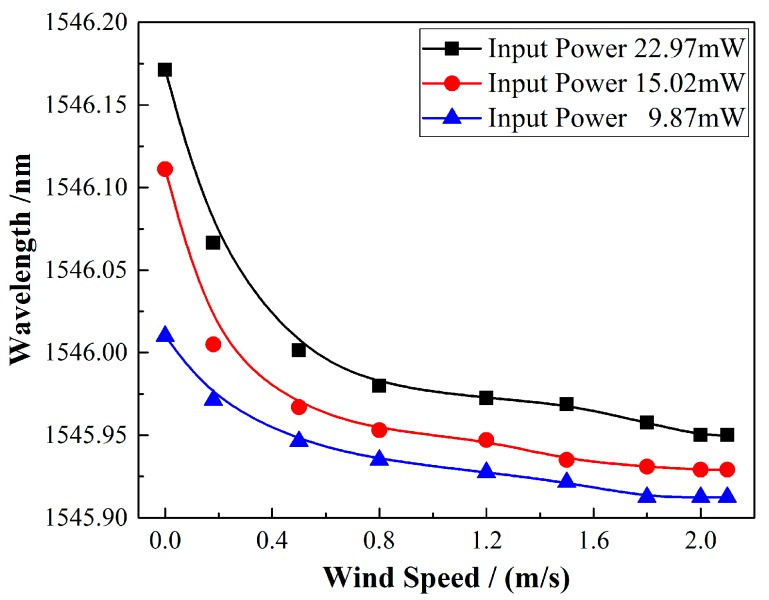
Wavelength responses versus the wind speed of the anemometer under different input powers.

**Figure 11:**
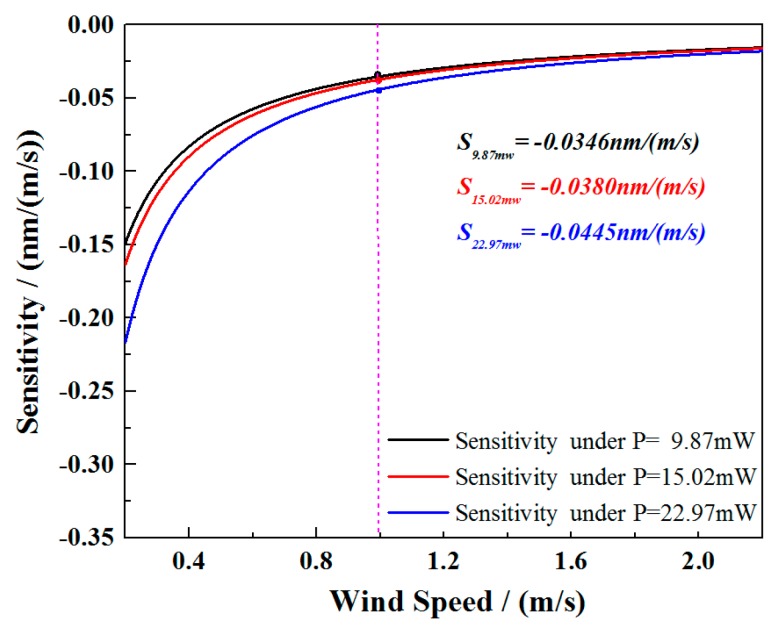
Sensitivity as a function of wind speed under different input power.

With the optimized parameters of the TFBG and SWCNT film, the investigation of the low power consumption of the system was conducted. A 12° TFBG with a 1.6 μm SWCNT film was selected to measure the wavelength responses under the input power from 22.97 down to 9.87 mW. Figure 10 shows the sensor responses with different input powers. As seen from the picture, the monitored wavelengths under the same 2.0 m/s wind speed were different. This could be explained by the differences in heat balance temperature of the sensor head under different pumping powers. Because the response of the sensor is not linear, in Figure 11, the first derivatives of the fitted curves are used to estimate the sensitivity of the sensor, and the sensitivities at wind speed ν = 1 m/s are −0.0346 nm/(m/s), −0.0380 nm/(m/s), and −0.0445 nm/(m/s) under the input powers of 9.87 mW, 15.02 mW, and 22.97 mW, respectively. If a common commercially available wavelength interrogation device with a 1 pm resolution and precision is applied in the system instead of an OSA with a relatively low wavelength resolution, the resolution of the sensing system can be 0.029 m/s, 0.026 m/s, and 0.023 m/s under each input power level. It is worth noting that our optimized sensor still worked well and showed a good wavelength response of 0.100 nm under an input power of less than 10 mW. The wavelength response decreases slowly as the wind speed grows. At the maximum speed of 2.0 m/s, the response reaches to its limit due to the thermal balance of the sensor head under such input power. Compared with the most recent report of a low-power-consumption fiber anemometer [20], our proposed sensing system sacrifices the wavelength response within an acceptable degree, but reveals a significant low-cost advantage with the simple design both in the manifesting process of the sensor and the establishment of the whole system. In addition, the reported system required a pumping laser, a specially designed MOF as well as an FBG integrated with a BBS (working under an uncommon wavelength band) to achieve the lower energy-consuming purpose. However, for the system proposed in this work, only a common low power BBS and a TFBG working in the optical communication band are used, which make it competitive for future remote long-term wind flow sensing and on-chip applications. 

## 4. Conclusions

In this paper, a simplified fiber-optic hot-wire anemometer with low power consumption based on an SWCNT-coated TFBG was proposed and demonstrated. Through the TFBG, the light in the core can be coupled into the cladding of the fiber and strongly absorbed by the SWCNT film deposited on the fiber surface. When the SWCNT film is heated up, the speed of wind flow around the HWA determines the cooling rate of the film and can be deduced from the spectrum of TFBG. Aimed at the low-power-consumption fiber-optic anemometer, we fully investigated and optimized the sensor’s key parameters including the inherent angle of the TFBG, the thickness of the SWCNT film, and the input power. The experimental results showed that the anemometer had better performances with an increased film thickness and an inherent angle of the TFBG. In the experiments, the temperature of the local area on the fiber was heated up to 46.6 °C under an input power of 22.97 mW and the sensitivity at wind speed ν = 1 m/s was found to be −0.0445 nm/(m/s). More importantly, taking advantage of both a TFBG and SWCNTs, with an optimized 12° TFBG and a 1.6 μm SWCNT film, the sensor is able to work under a very low input power of only 9.87 mW. Another distinguished advantage of our proposed sensing system is that only one common low-power BBS was needed as both sensing and heating light source, which greatly simplified the system and reduced the cost. Therefore, this proposed simple and low-cost sensing system provides a promising platform for future long-term remote flow sensing and on-chip applications.
